# Takayasu arteritis in childhood: retrospective experience from a tertiary referral centre in the United Kingdom

**DOI:** 10.1186/s13075-015-0545-1

**Published:** 2015-02-25

**Authors:** Despina Eleftheriou, Giulia Varnier, Pavla Dolezalova, Anne-Marie McMahon, Muthana Al-Obaidi, Paul A Brogan

**Affiliations:** UCL Institute of Child Health, 30 Guilford Street and Great Ormond Street Hospital for Children NHS Foundation Trust, London, WC1N1EH UK; Department of Paediatric Rheumatology, Great Ormond Street Hospital for Children NHS Foundation Trust, London, UK; Department of Paediatrics and Adolescent Medicine, Paediatric Rheumatology Unit, Charles University in Prague and General University Hospital in Prague, Prague, Czech Republic; Department of Paediatric Rheumatology, Sheffield Children’s Hospital, Sheffield, South Yorkshire S10 2TH UK

## Abstract

**Introduction:**

Takayasu arteritis (TA) is an idiopathic large-vessel vasculitis affecting the aorta and its major branches. Although the disease rarely affects children, it does occur, even in infants. The objective of this study was to evaluate the clinical features, disease activity, treatment and outcome of childhood TA in a tertiary UK centre.

**Methods:**

We analysed a retrospective case series of children fulfilling the TA classification criteria of the European League against Rheumatism, the Paediatric Rheumatology European Society and the Paediatric Rheumatology International Trials Organisation. Data regarding demographics, clinical features, treatments and outcomes were recorded. Descriptive statistics are expressed as median and range. Fisher’s exact test was used for group comparisons. The Paediatric Vasculitis Activity Score (PVAS), Paediatric Vasculitis Damage Index (PVDI), Disease Extent Index-Takayasu (DEI.Tak) and Indian Takayasu Arteritis Activity Score (ITAS2010) were calculated retrospectively.

**Results:**

A total of 11 children (64% female) with age at diagnosis of 11.8 (1.3 to 17) years were identified over a 23-year period. The median time to diagnosis was 17 (0 to 132) months. The most common clinical features at presentation were arterial hypertension (72.7%), systemic features (36%) and cardiovascular (45%), neurological (36%), pulmonary (27%), skin (9%), renal (9%) and gastrointestinal (9%) involvement. At presentation, PVAS was 5/63 (1 to 13); DEI.Tak was 7/81 (2 to 12) and ITAS2010 was 9/57 (6 to 20). Treatment included corticosteroids (81.8%), combined with methotrexate in most cases (72.7%). Cyclophosphamide (36.4%) and biologic agents (45.5%) were reserved for severe and/or refractory cases. PVDI at latest follow-up was 5.5/72 (3 to 15). Mortality was 27%. Young age at disease onset (<5 years old) and permanent PVDI scores ≥3 were significantly associated with mortality risk (*P* = 0.024).

**Conclusion:**

TA is a rare and potentially life-threatening large-vessel vasculitis. Improved awareness of TA is essential to secure a timely diagnosis. Although the evidence base for the treatment of TA in children is weak, we found that it is essential to treat it aggressively because our data emphasise that the mortality and morbidity in the paediatric population remains high.

**Electronic supplementary material:**

The online version of this article (doi:10.1186/s13075-015-0545-1) contains supplementary material, which is available to authorized users.

## Introduction

Takayasu arteritis (TA) is an idiopathic large-vessel vasculitis (LVV) affecting the aorta and its major branches [1]. TA has a worldwide distribution, with a reported incidence of 1.2 to 2.6 per 1 million per year in Caucasians and a 100-fold higher incidence in East Asians [2-4]. Although the disease rarely affects children, it does occur, even in infants [5-7]. Diagnostic delay is unfortunately common [5], sometimes occurring years after the acute onset of TA. Untreated TA commonly leads to cardiovascular injuries, such as aneurysm formation, concentric arterial wall fibrosis and thrombotic complications [8,9]. Because large artery biopsies are usually infeasible and laboratory investigations are neither sensitive nor specific, the diagnosis of TA is based on clinical features, including a history compatible with systemic inflammation (with or without documentation of acute-phase response) and angiographic features compatible with LVV [7,8]. *Angiography* refers to either formal catheter, computed tomography angiography (CTA) or magnetic resonance angiography (MRA) [10,11]. Other imaging modalities, such as ^18^F-fluorodeoxyglucose (^18^F-FDG) positron emission tomography-computed tomography (PET-CT) [12] or, more recently, ^18^F-FDG PET magnetic resonance imaging (^18^F-FDG-PET-MRI) [10] can provide important adjunctive information, although their sensitivity and specificity for TA disease activity are not well defined, particularly in children.

Notably, there is a lack of high-quality evidence on which to base treatment recommendations in adults, and there is even less in children [13]. Hence, there is an ongoing need to describe the limited paediatric TA experience available because formal clinical trials are unlikely to come to fruition within the next decade, if ever. The majority of currently published studies describe childhood-onset disease in countries where TA is most prevalent [7,11,14]. The clinical phenotype and disease burden in the UK paediatric population are currently not well defined, even though case reports have raised concerns that TA may have the worst long-term prognosis of all the paediatric vasculitides in the United Kingdom [5,8,9]. To address this issue, we established the following aims in the present study: (1) to describe the presenting clinical, histopathological and radiological findings; (2) to explore the utility of currently available disease activity and damage instruments for evaluating TA; (3) to describe the treatments used; and (4) to describe outcomes for children with TA in a UK paediatric rheumatology tertiary referral centre.

## Methods

### Patient identification and ethical approval

We identified, by searching clinical and radiological databases, all the patients with a clinical diagnosis of TA seen at Great Ormond Street Hospital (GOSH) in London between January 1990 and May 2013. One patient was referred to GOSH from Sheffield Children’s Hospital; the team there (AM and MO) continued to provide shared care for this child. Case notes were reviewed retrospectively, and patients fulfilling the following classification criteria of the European League against Rheumatism, the Paediatric Rheumatology European Society and the Paediatric Rheumatology International Trials Organisation for childhood TA were included in the study [15]: angiographic abnormalities of the aorta or its main branches (mandatory criterion) plus at least one of the following four features—(1) decreased peripheral artery pulse(s) and/or claudication of extremities; (2) blood pressure difference >10 mmHg; (3) bruits over the aorta and/or its major branches; and/or (4) hypertension (related to childhood normative data) [15]. Ethical approval was granted by the Institute of Child Health/GOSH Ethics Committee for a retrospective case notes review, and no individual patient consent was therefore required.

### Data gathered

Data recorded at diagnosis included sex, age, ethnicity, blood pressure and organ involvement. Disease activity was assessed retrospectively using a number of tools: the Paediatric Vasculitis Activity Score (PVAS) [16], the Disease Extent Index-Takayasu (DEI.Tak) [17] and the Indian Takayasu Arteritis Activity Score (ITAS2010) (Additional files 1, 2, 3, 4 and 5) [18]. The PVAS is a validated disease activity tool specific to paediatric vasculitis and is the only tool currently used as an outcome measure in paediatric vasculitis clinical trials. Despite that, data related to PVAS and LVV are limited. The ITAS2010 and DEI.Tak are TA-specific disease activity tools. The DEI.Tak was designed to record disease extent, including both activity and damage. The ITAS2010 provides a quantitative score of new active disease. The use of these two TA-specific tools has been validated in large adult studies for interrater reliability, convergence with Birmingham Vasculitis Activity Score (BVAS), correlation with the Physician’s Global Assessment and erythrocyte sedimentation rate (ESR) and serum C-reactive protein (CRP), ability to discriminate between active and inactive disease state at first visit, and sensitivity to change. Neither of the two TA-specific tools is currently used in routine paediatric practice, however. Therefore, the purpose of using all three in the present study was to compare and contrast them to provide insight into their relative clinical utility and to inform future studies regarding their refinement. ESR, serum CRP, cardiac enzymes (troponin where available), levels of antineutrophil cytoplasmic antibodies (ANCA) and antinuclear antibodies (ANA), and histopathological findings were reviewed. Other investigation results recorded, when available, included electrocardiograms (ECGs), echocardiograms, plain radiographs, ultrasonograms, CTA scans, MRA and MRI scans, digital subtraction angiography (DSA) scans, ^18^F-FDG-PET scans and dual-energy x-ray absorptiometry (DEXA) scans. The time to diagnosis and medical and surgical treatments were also recorded. Outcome measures included changes in acute-phase reactants, PVAS, ITAS2010, DEI.Tak, z-scores for height and weight at latest follow-up, cause of death and contributing factors, disease and treatment-related adverse events, and frequency of osteopenia, defined as a z-score for bone density more than 2 standard deviations below the mean for age identified with a DEXA scan. The Paediatric Vasculitis Damage Index (PVDI) [19] was calculated retrospectively. PVDI is based on a modified version of the adult Vasculitis Damage Index tool [19]. Despite the fact that the paediatric version is not yet validated, we explored its utility in the present study because it provides a systematic approach to the documentation of damage in the context of vasculitis.

### Statistical analysis

Continuous variables were summarized as median and range. Categorical variables are presented as percentages and frequencies. Parameters between groups were compared using Fisher’s exact test. Wilcoxon matched-pairs signed-rank tests were used for comparisons before and after biologic therapy. Correlations between disease activity tools were assessed using Spearman’s correlation coefficient. A *P*-value (two-sided) <0.05 was considered significant. Statistical analyses were performed using IBM SPSS version 18 software (IBM, Armonk, NY, USA). Growth standard deviation scores (z-scores) were calculated based on UK normative data using a free downloadable Microsoft Excel add-on [20].

## Results

### Demographics and clinical manifestations at presentation

A total of 11 children (64% female, n = 7) aged 11.8 (1.3 to 17.0) years who fulfilled the classification criteria for TA were identified (Table 1). Eight (72%) of eleven were Caucasian, two (18%) were Asian and one (9%) was Afro-Caribbean. The median interval from first symptom onset to diagnosis was 17 (0 to 132) months. PVAS at presentation was 5/63 (1 to 13); DEI.Tak was 7/81; and ITAS2010 was 9/57 (6 to 20). The classification of TA was based on the presence of angiographic abnormalities of the aorta or its main branches (present in all 11 patients), plus at least one of the following four features: decreased peripheral artery pulse(s) (3/11, 27%), blood pressure difference >10 mmHg (3/11, 27%), bruits over the aorta and/or its major branches (6/11, 54%) and/or hypertension related to childhood normative data (8/11, 72%).

**Table 1:** **Time to diagnosis, disease activity measures and outcomes in a cohort of 11 patients with childhood Takayasu arteritis** ^**a**^

**Patient number**	**Time to diagnosis (mo)**	**PVAS/ITAS2010/DEI.Tak at diagnosis**	**Surgical/endovascular intervention(s)**	**Follow-up (mo)**	**Status at last follow-up**
1	42	1/6/5	Repair of LV aneurysm (twice), ascending aorta aneurysm repair, AMPLATZER Septal Occluder device (AGA Medical, Golden Valley, MN, USA) insertion, stent insertion within ascending aorta, coil embolisation of right CA aneurysm	11	Remission on treatment
2	2	10/15/12	Mitral valvuloplasty and aortic root replacement, subsequent LIMA bypass graft for stenotic left CA	32	Remission on treatment
3	4	5/16/7	–	16	Relapse on treatment
4	7	10/15/9	–	36	Remission on treatment
5	132	2/6/2	–	24	Remission off treatment
6	0	2/10/6	Angioplasty of both renal arteries, SMA and external iliac arteries; coil embolisation of a cerebral artery aneurysm	168	Deceased
7	120	5/9/7	–	11	Deceased
8	17	3/8/8	–	6	Remission on treatment
9	18	1/6/5	–	6	Remission off treatment
10	0	3/10/5	Angioplasty of the renal arteries (twice)	26	Remission on treatment
11	60	12/9/12	Angioplasty of abdominal aorta; required subsequent surgical repair	14	Deceased

^a^CA, Coronary artery; DEI.Tak, Disease Extent Index-Takayasu; ITAS2010, Indian Takayasu Arteritis Activity Score; LIMA, Left internal mammary artery; LV, Left ventricle; PVAS, Paediatric Vasculitis Activity Score; SMA, Superior mesenteric artery. Total possible scores range from 0 to 63 for PVAS, from 0 to 57 for ITAS2010 and from 0 to 81 for DEI.Tak (0–81). Higher scores reflect higher disease activity for all tools [16-18].

The main presenting clinical features are shown in Table 2. Arterial hypertension was the commonest presenting feature.

**Table 2:** **Presenting clinical features in a UK-based cohort of 11 patients with childhood Takayasu arteritis** ^**a**^

**Presenting features**	**Number of patients with features (%)**
Sex	
Female:male	7 (64%):4 (36%)
Ethnicity	
Caucasian	8 (73%)
Asian	2 (18%)
Afro-Caribbean	1 (9%)
Systemic features	4 (36%)
Fever	4 (36%)
Weight loss >5% of body weight	4 (36%)
Myalgia	1 (9%)
Arthralgia or arthritis	1 (9%)
Skin involvement	1 (9%)
Livedo reticularis	0 (0%)
Purpura	1 (9%)
Other skin vasculitis (vasculitis different from previous, such as subcutaneous oedema, Raynaud’s phenomenon)	1 (9%)
Mucous membranes/eyes	1 (9%)
Red eye conjunctivitis	1 (9%)
Arterial hypertension >95% percentile for age	8 (73%)
Renal involvement	1 (9%)
Proteinuria (>0.3 g/24 hr or >20 mmol/mg creatinine)	1 (9%)
Haematuria (≥5 rbc/hpf or rbc casts)	1 (9%)
Rise in creatinine >10% or creatinine clearance (GFR) decrease >25%	1 (9%)
Neurological involvement	4 (36%)
Headache	4 (36%)
Organic confusion/cognitive dysfunction	1 (9%)
Meningitis/encephalitis	0 (0%)
Seizures (not hypertensive)	2 (18%)
Stroke	2 (18%)
Gastrointestinal involvement	1 (9%)
Abdominal pain	1 (9%)
Peritonitis	0 (0%)
Blood in the stools or bloody diarrhoea	0 (0%)
Bowel ischaemia/perforation	0 (0%)
Cardiovascular involvement	5 (45%)
Loss of pulses	2 (18%)
Bruits over accessible arteries	5 (45%)
Blood pressure discrepancy	2 (18%)
Claudication of extremities	1 (9%)
Ischaemic cardiac pain	2 (18%)
Cardiomyopathy	3 (27%)
Congestive cardiac failure	2 (18%)
Valvular heart disease	1 (9%)
Pericarditis	1 (9%)
Pulmonary involvement	3 (27%)
Wheeze or expiratory dyspnoea	3 (27%)
Pleural effusion	3 (27%)
Infiltrate	2 (18%)
Massive haemoptysis/alveolar haemorrhage	0 (0%)
Respiratory failure	3 (27%)

^a^GFR, Glomerular filtration rate; hpf, High-powered field; rbc, Red blood cells.

Established cardiac and/or large arterial injury was common at presentation. A total of 5 (45%) of 11 patients presented with bruits over the aorta and/or its major branches. Three patients (27%) had cardiomyopathy. Loss of peripheral pulses was documented in two patients (18%). Ischaemic cardiac pain was confirmed on ECGs and with elevated troponin in two patients (18%). Lower-limb claudication was present in one patient (9%), pericarditis in one patient (9%) and valvular heart disease in one patient (9%).

Two patients had major cardiac involvement at initial presentation. Patient 1 presented with a giant aneurysm of the left ventricle (LV). The aneurysm was considered traumatic because his symptoms were preceded by direct (albeit mild) trauma to his chest, and he underwent surgical repair. Over the course of the next 4 years, he had a number of surgical procedures, including surgery for LV aneurysm recurrence. Histology of the LV and aortic wall tissue indicated the presence of inflammatory cells and laminated thrombus compatible with a false aneurysm, and the aortic wall intima showed necrosis with surface fibrinous exudate. On the basis of these cardiac histological findings, he was eventually diagnosed with TA. Patient 2 presented in infancy, when she was admitted acutely to the cardiac intensive care unit with pulmonary oedema secondary to severe acute aortic and mitral valve regurgitation; the full details of this case are published elsewhere [5].

### Laboratory parameters

At diagnosis for all 11 patients, the median ESR was 72 (12 to 108) mm/hr and CRP was 53 (0 to 237) mg/L. ANCA were negative for four patients tested. ANA were modestly positive (≤1:320) in two of six patients tested. For two (18%) of eleven patients who presented with ischaemic cardiac pain, there was an increase in troponin levels 8.7 (range 5.40-12; normal levels <0.04 microgram/L).

### Histology

Histology results were available for five (45%) of eleven patients. Four were obtained at diagnosis: mitral valve (n = 1), left ventricle myocardium (n = 1), pericardium (n = 1) and intestinal biopsy (n = 1). One was obtained from a postmortem autopsy for one of the deceased patients (renal and cardiac tissue; patient 7). Histology showed florid vasculitis in three of the five patients, fibrous intimal thickening in four patients (periadventitial and adventitial), calcification in one, small vessel thrombosis in two, granulomas in one and neovascularisation in two. Intestinal biopsy was normal in the one single patient that had an intestinal biopsy.

### Imaging studies

MRI with gadolinium contrast enhancement and/or MRA imaging studies (performed in all patients at diagnosis) revealed abnormalities in all patients as follows: arterial narrowing and stenosis (10/11; 90%), arterial dilatation or aneurysm formation (7/11; 63%), arterial wall thickening (9/11; 81%) and collateral vessels or other calibre variations (10/11; 90%). A total of eight (73%) of eleven patients had positive gadolinium contrast arterial wall enhancement, consistent with active LVV at the time the study was undertaken. CTA was performed in six patients, which revealed pseudoaneurysms and/or aneurysms (4/6), reduced and/or absent blood flow (3/6), narrowing and/or stenosis (4/6), arterial calcifications (2/6) and occlusion (1/6). DSA was performed in four (36%) of eleven patients and showed stenosis of thoracic and abdominal aortas in two patients, collaterals in two, stenosis of renal arteries in one and cerebral aneurysms in one. ^18^F-FDG-PET was performed in four (36%) of eleven patients and revealed avid uptake, mostly in the ascending aorta, in two of four studies.

The distribution of involved arteries according to the angiographic classification of TA [21] showed that the most common type of vessel involvement was type V (Table 3). The most common type of lesion was stenosis, which was present in nine (81%) of eleven patients, followed by aneurysms in five (45%), dilatation in three (27%) and occlusion in two (18%).

**Table 3:** **Distribution of involved vessels according to the new angiographic classification of Takayasu arteritis in a cohort of 11 children in a UK-based centre** ^**a**^

**Type**	**Types of affected vessels**	**Number of patients (N = 11)**
I	Branches from aortic arch only	0
IIa	Ascending aorta, aortic arch and its branches	1 C+
IIb	Ascending aorta, aortic arch and its branches, thoracic descending aorta	0
III	Thoracic descending aorta, abdominal aorta and/or renal arteries	1 P+
IV	Abdominal aorta and/or renal arteries	2
V	Combined features of types IIb and IV	7 (1/7 C+)

^a^According to this classification system [21], involvement of the coronary or pulmonary arteries is designated as C(+) or P(+), respectively.

### Treatment

The general therapeutic approach in our cohort was that of induction of remission (high-dose corticosteroid combined with another immunosuppressant), followed by maintenance of remission therapy (lower-dose corticosteroid combined with a maintenance immunosuppressive agent, usually methotrexate (MTX)) or institution of second-line therapy for failed induction. The medical treatments received by the patients are summarized in Additional file 6: Table S1.

#### Corticosteroids

Nine (81%) of eleven patients received corticosteroids as induction therapy. Five of these nine patients received prednisolone at 1 or 2 mg/kg/day; the remaining four received intravenous methylprednisolone 30 mg/kg for 3 days and were then switched to oral prednisolone 1 or 2 mg/kg/day. Two patients (patients 5 and 7) did not receive corticosteroids (or any other immunosuppressive agent), because the diagnosis of TA was made in the late stenotic phase of the disease (132 and 123 months, respectively, after the acute onset of the disease), and clinically and/or radiologically these patients had absence of disease activity.

#### Adjunctive immunosuppressive therapy

First-line therapies were as follows: Four (36%) of eleven patients received weekly subcutaneous MTX combined with corticosteroids three (27%) received cyclophosphamide at induction, two (18%) received intravenous immunoglobulin (2 g/kg) and one (9%) received azathioprine (2 to 3.7 mg/kg/day).

Regarding second-line therapy, five (45%) of eleven patients were switched to MTX at a median of 8 (5 to 132) months from diagnosis. Overall, there was evidence of ongoing disease activity at that time as indicated by all three disease activity measures: PVAS of 7/63 (2 to 11), ITAS2010 of 11/57 (5 to 14/), DEI.Tak of 8/81 (8 to 15/81). At that time point, median ESR was 11 (2 to 56) mm/hr and median CRP was 9.5 (1 to 20) mg/L. The median dose of oral prednisolone for these patients was 0.3 (0.2 to 0.75) mg/kg/day. Patient 11, who had been on MTX for 7 months as a first-line therapy, was switched to cyclophosphamide because she had progressive disease detected by MRA.

#### Biologic therapies

Biologic therapy was initiated for 6 (54%) of 11 patients at 11 (4 to 156) months from diagnosis. The median ESR was 63 (3 to 115) mm/hr, and the median CRP was 37 (3 to 140) mg/L. Anti–tumour necrosis factor alpha (anti-TNFα) therapy was the first class of biologic agent chosen in all six cases. Three patients received adalimumab (24 mg/m^2^ fortnightly if body weight was <30 kg, 40 mg fortnightly if >30 kg) as the first biologic. Three patients received infliximab (6 mg/kg intravenously monthly) as the first biologic, including patient 2, who was later converted to adalimumab because of difficulties with venous access. Patient 3 was switched from adalimumab to tocilizumab therapy at 14 months from diagnosis because he developed severe myalgia and arthralgia with raised inflammatory markers. The median dose of prednisolone at time of biologic therapy initiation was 0.2 (0.1 to 2) mg/kg/day, and, at a median of 2.5 (2 to 28 months) of biologic therapy, it was 0 (0 to 0.6) mg/kg/day (*P* = 0.41). In addition, in response to this biologic therapy, PVAS changed from 9.5/63 (4 to 24) to 0/63 (0 to 21) (*P* = 0.312), ITAS2010 changed from 6/57 (4 to 18) to 0/57 (0 to 18) (*P* = 0.104) and DEI.Tak changed from 3/81 (1 to 20) to 0/82 (0 to 20) (*P* = 0.187). Of note, in patient 3, the PVAS score was 0/63 at the time of initiation of biologic therapy, indicating absence of clinical disease activity; however, the ITAS2010 and DEI.Tak scores were 4/57 and 2/81, respectively. Regarding patient 3, following an initially promising response to tocilizumab, the disease relapsed 12 months later, and, at the time of this writing, this patient was receiving rituximab.

#### Other medical therapy

Additional file 6: Table S1 (available online) summarizes the adjunctive medical therapy received. Seven (63%) of eleven patients required a median of three (range, from two to six) antihypertensive medications, five received antiplatelet doses of aspirin and two received anticoagulation (warfarin after initial heparinisation). Patient 11 required treatment with sildenafil and epoprostenol infusion for pulmonary arterial hypertension that developed 4 months following diagnosis.

#### Surgical and endovascular management

Five patients required surgical and/or endovascular treatment. These procedures are summarized in Table 1.

### Outcomes

The median length of follow-up was 16 (6 to 168) months. At the last follow-up, PVAS was 0/63 (0 to 21), ITAS2010 was 0/57 (0 to 18) and DEI.Tak was 0/81 (0 to 20). ESR was 5 (2 to 20) mm/hr, and CRP was 5 (range 1 to 12) mg/L. Seven of eleven patients were in remission at latest follow-up, as indicated by normal CRP and ESR values and a PVAS of 0 on two consecutive measurements 1 month apart [16]. The following corticosteroid-associated side effects were documented in 72.7% (8/11) of the patients: Cushing’s syndrome (5/8; 62%), osteoporosis (2/8; 25%), skeletal fracture (1/8; 12.5%) and hip avascular necrosis (1/8; 12.5%).

The median z-score for height at diagnosis was −0.10 (−1.39 to 3.79), and at the last follow-up it was −0.89 (−5.6 to 1.58) (*P* = 0.019). The median z-score for weight at diagnosis was +1.13 (−0.96 to 3.46), and it was +0.47 (−9.21 to 2.57) at the last follow-up (*P* = 0.019).

Two patients had pancytopenia, which was related to treatment with MTX and cyclophosphamide, respectively. Both recovered with cessation of therapy. Patient 11 developed *Candida* and *Pseudomonas* sepsis whilst in intensive care for multiorgan failure.

The median PVDI at latest follow-up was 5.5/72 (3 to 15). When the items that had been present for more than 3 months at one time but had resolved at latest follow-up [19] were removed, the PVDI changed to 3/72 (0 to 10).

Three of eleven patients died during follow-up (27% mortality). Patient 7 presented with acute cardiomyopathy, and no clear diagnosis was made at initial presentation. Approximately 6 years later, the diagnosis of TA was made based on the loss of peripheral pulses and lower-limb claudication. Patient 7 did not receive any immunosuppressive treatment, as he was considered to be in the late stenotic phase of the disease. Four years after receiving his diagnosis, he collapsed suddenly and died. The postmortem examination revealed severe LV hypertrophy and 70% occlusion of the coronary arteries. His abdominal aorta was narrowed and had a patchy and sparse chronic inflammatory cell infiltrate and evidence of active inflammation within the vasa vasorum of the distal aorta. His final PVDI was 5/72.

Patient 6 was diagnosed with TA at screening after her sister had died as a result of TA [9]. Patient 6 received treatment with corticosteroids, azathioprine, MTX and adalimumab. She accrued significant damage over time. Her final PVDI score was 10/72. She died suddenly as a result of acute aortic rupture 15 years following diagnosis.

Patient 11 was diagnosed with coarctation of the aorta during her first 5 years of life and had undergone surgical repair with patch augmentation. She was followed up for severe arterial hypertension for the next 3 years. At 3 years after her diagnosis, she presented with acute respiratory failure, pericarditis, no palpable pulses on the lower limbs due to critical stenosis of the whole aorta and the main branches, anuria and hypertension. She died as a result of progressive multiorgan failure, despite receiving escalating immunosuppression (high-dose intravenous methylprednisolone, MTX, cyclophosphamide, infliximab). Her final PVDI was 12/72.

### Correlations between Takayasu arteritis vasculitis disease activity tools

**Figure 1:**
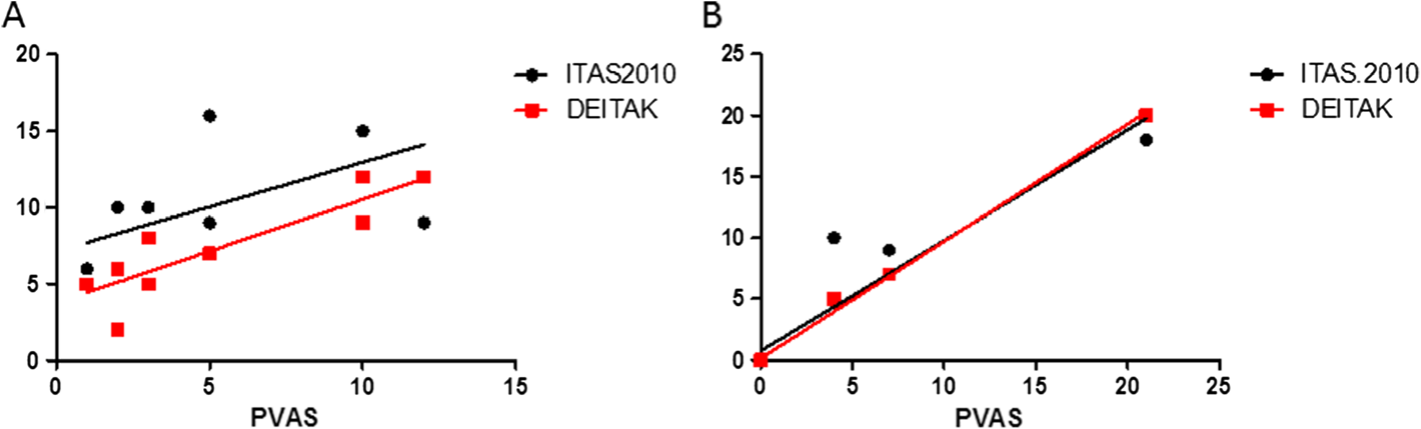
**Correlations between Takayasu arteritis disease activity measure tools. (A)** Correlations at time of diagnosis. **(B)** Correlations at time of latest follow-up. DEI.Tak, Disease Extent Index-Takayasu; ITAS2010, Indian Takayasu Arteritis Activity Score; PVAS, Paediatric Vasculitis Activity Score. Total possible scores: PVAS: 0 to 63, ITAS2010: 0 to 57 and DEI.Tak: 0 to 81. Higher scores reflect higher disease activity for all tools [16-18].

Figure 1 shows the correlations among the various TA disease assessment instruments at the time of diagnosis and follow-up. PVAS correlated strongly with both ITAS2010 (*r* = 0.66; *P* = 0.030) and DEI.Tak (*r* = 0.86; *P* = 0.0012) at diagnosis and latest follow-up (ITAS2010: *r* = 0.98, *P* = 0.0001; DEI.Tak: *r* = 1, *P* = 0.0001).

### Factors associated with mortality

Age at disease onset ≤5 years was significantly associated with mortality (*P* = 0.024). Permanent damage items on PVDI scoring at latest follow-up ≥3/72 were also significantly associated with mortality (*P* = 0.024). There was no statistical association between mortality and time to diagnosis >12 months (*P* = 0.545), cardiac involvement (*P* = 0.18), endovascular procedures within 12 months of diagnosis (*P* = 0.545) or need for biologic therapy (*P* = 1) (Additional file 7: Table S2).

## Discussion

In our present retrospective study, we report the largest UK cohort so far of paediatric patients with TA. This study is the first to systematically apply three different vasculitis disease activity scores and to explore damage accrual using the PVDI. We observed a significant delay in diagnosis (up to 132 months) due to the insidious and non-specific presentation of TA. The burden of disease and its therapy was high, as depicted by PVDI scores at latest follow-up. Biologic therapies were used in 54% of patients for therapy-resistant disease. Mortality was high at 27%. Disease onset before 5 years of age and persistent damage items providing PVDI scores ≥3 were significantly associated with mortality.

Similarly to other case series, the most common presenting feature of TA in our study was arterial hypertension (70%), but notably in the absence of other systemic symptoms, which may partly explain the diagnostic delay [6-8,14]. We report a higher frequency of cardiac and neurological involvement in our sample compared with other published paediatric TA cohorts, perhaps reflecting the severity of the cases referred to our tertiary care centre. The frequency of cutaneous and musculoskeletal symptoms were similar to other reports, however [6-8,14]. We emphasize that severe cardiac involvement can be the first presenting feature of LVV in children (3 of 11 patients in our study).

Histology for the majority of our cases revealed bland lymphocytic inflammation with some neovascularisation, but absence of granulomas, in contrast to histological studies of adult-onset TA, where granulomas are often a major feature [22]. Because mononuclear infiltration of the adventitia with perivascular cuffing of the vasa vasorum is common in the early phase of TA [22,23], we suggest that one reason why granulomas may not be so conspicuous in children is that these patients are likely to have had shorter disease duration at the time of tissue sampling.

**Figure 2:**
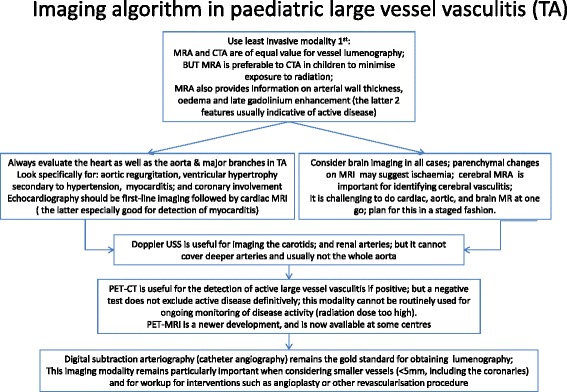
**Recommended imaging algorithm for children with Takayasu arteritis.** CT, Computed tomography; CTA, Computed tomography angiography; MRA, Magnetic resonance angiography; MRI, Magnetic resonance imaging; PET, Positron emission tomography; TA, Takayasu arteritis; USS, Ultrasound.

Assessment of disease activity in TA remains challenging, particularly in children [8]. Acute-phase reactants lack sufficient sensitivity to be clinically reliable; most clinical manifestations are non-specific, and vascular injury progresses insidiously [8]. Active vasculitis may still be present, even in the absence of an acute-phase response and with negative ^18^F-FDG PET-CT scans as in patient 7, in whom autopsy results demonstrated chronic active LVV. On the basis of our experiences and those reported by others [10], we provide an approach to the use of imaging for the diagnosis and monitoring of TA in paediatric patients (Figure 2). In particular, MRI techniques coregistered with PET have the potential to improve evaluation of TA, both at diagnosis and during follow-up. The role of circulating biomarkers such as pentaxin 3 (PTX3) level is currently unclear [24], but PTX3 could have a role if validated in a clinical context.

Two specific tools to assess TA disease activity and extent have been validated in adults: ITAS2010 and DEI.Tak [17,18]. These tools were derived from the BVAS, but with particular emphasis on cardiovascular manifestations because these predominate in TA. Although they are not validated in children, we explored their utility in comparison with the PVAS, the only validated vasculitis activity score for children. Perhaps unsurprisingly, activity scores using both TA-specific tools were consistently higher than those with PVAS, as the former allow more detailed assessment of the extent of vascular involvement. We note, however, the strong correlation of measurements between the PVAS versus ITAS2010 and DEI.Tak tools at both diagnosis and follow-up, suggesting that, overall, all three tools consistently reflect TA disease activity. Development of these TA-specific tools to include modifications for age-specific items such as body weight and arterial hypertension, as well as prospective validation in children with TA, is now warranted. In addition, using standard definitions of remission employed in clinical trials for other vasculitides, a total of 7 of 11 patients fulfilled remission criteria at their latest follow-up examination. We acknowledge, however, that grumbling disease activity may not be sufficiently captured by these clinical indices. Further studies designed to refine and validate reliable definitions of disease remission and response to therapy in TA are now needed.

We also explored the utility of the prototypic PVDI [19] for the first time in an attempt to systematically assess damage accrual. All patients scored high for items related to cardiovascular morbidity, and 8 of 11 scored positively for treatment-related morbidity items. Notably, only permanent damage items detected using PVDI were associated with mortality.

Corticosteroids combined with MTX or cyclophosphamide were the standard induction therapy for our patients. More than one-half (54%) required addition of biologic therapy (within the latter 10 years of the study, when these treatments became available) because of a severe and refractory disease course. In addition to anti-TNFα agents, promising results have been reported with anti-interleukin-6 therapy (tocilizumab) for adults with TA [25-27]. Tocilizumab was used for one of our patients, who went on to receive rituximab for inadequate disease control. There has never been a controlled trial for the treatment of TA. Rare disease trial designs that have recently been adapted for other rare childhood vasculitides (for example, the open-label randomised controlled trial of mycophenolate mofetil versus cyclophosphamide for the induction of remission of childhood polyarteritis nodosa (ISRCTN 75434563)) should also be considered for children with TA [28].

We report a mortality rate of 27%, which is in line with mortality rates reported by others (ranging from 16% to 40%) [6,7,14]. Disease mortality was related to younger age at presentation (*P* = 0.024). This finding is unsurprising, given that these children had a prolonged, severe disease course and extensive stenotic disease well before adolescence. Whether these patients represent a distinct group of early-onset severe disease due to an as yet undefined genetic disorder remains to be established [9], and this is an area of ongoing study by our group.

Our study is limited by all the confounding factors associated with any retrospective case series. We acknowledge the small sample size and lack of heterogeneity of our cohort. There is also a possibility that referral bias resulted in the more severe spectrum of the disease observed in our patients.

## Conclusions

TA in children is a rare but potentially life-threatening condition. Improved awareness of TA is essential to securing an early diagnosis. LVV should be considered in any young child presenting with hypertension with or without elevated acute-phase reactants. Other clinical features are unfortunately non-specific, but the case for suspected TA is even stronger in a patient with hypertension in the presence of fever, weight loss, headaches, arthralgia, fatigue and/or bruits. In the future, use of the currently available clinical scores explored herein may be beneficial for monitoring response to therapy. Undoubtedly, however, imaging is particularly important for the diagnosis and monitoring of LVV (Figure 2). Although the evidence base for the treatment of TA in children is weak, it is essential to treat TA aggressively as soon as the diagnosis is secured to reduce mortality and morbidity.

## Additional files

Additional file 1:
**Disease Extent Index for Takayasu Arteritis tool.**
Additional file 2:
**Indian Takayasu Arteritis Activity Score tool.**
Additional file 3:
**Indian Takayasu Arteritis Activity Score tool glossary.**
Additional file 4:
**Paediatric Vasculitis Activity Score tool.**
Additional file 5:
**Paediatric Vasculitis Activity Score tool glossary.**
Additional file 6: Table S1. Treatment for 11 children with Takayasu arteritis in a UK-based centre. †In case of failed induction. Pred = oral prednisolone; mepred = methylprednisolone (30 mg/kg/day intravenously three times per day, unless stated otherwise); MTX = methotrexate (15 mg/m^2^wk, unless otherwise stated); CYC = cyclophosphamide; iv = intravenous; sc = subcutaneous; IVIG = intravenous immunoglobulin; PVAS = Paediatric Vasculitis Activity Score; DEI.TAK = Disease Extent Index-Takayasu; ITAS2010 = Indian Takayasu Arteritis Activity Score 2010; asp = aspirin (2 to 5 mg/kg/day); inflix = infliximab (6 mg/kg intravenously monthly unless stated otherwise); adalim = adalimumab (24 mg/m^2^; maximum dose 40 mg, sc every 2 weeks); NA = not applicable. For other treatments where dose is not stated, standard paediatric doses were used. Additional file 7: Table S2. Factors associated with mortality in a cohort of 11 children with Takayasu arteritis seen in a UK tertiary referral centre. Fisher’s exact test was used for comparison between groups. PVDI = Paediatric Vasculitis Damage Index [19]. *P*-values <0.05 (two-sided) were considered significant.

## Abbreviations

^18^F-FDG: ^18^F-fluorodeoxyglucose
ANA: Antinuclear antibodies
ANCA: Antineutrophil cytoplasmic antibodies
BVAS: Birmingham Vasculitis Activity Score
CA: Coronary artery
CRP: C-reactive protein
CT: Computed tomography
CTA: Computed tomography angiography
DEI.Tak: Disease Extent Index-Takayasu
DEXA: Dual-energy X-ray absorptiometry
DSA: Digital subtraction angiography
ECG: Electrocardiogram
ESR: Erythrocyte sedimentation rate
GFR: Glomerular filtration rate
GOSH: Great Ormond Street Hospital
hpf: High-powered field
ITAS2010: Indian Takayasu Arteritis Activity Score
LIMA: Left internal mammary artery
LV: Left ventricle
LVV: Large-vessel vasculitis
MRA: Magnetic resonance angiography
MRI: Magnetic resonance imaging
MTX: Methotrexate
PET: Positron emission tomography
PVAS: Paediatric Vasculitis Activity Score
PVDI: Paediatric Vasculitis Damage Index
RBC: Red blood cells
SMA: Superior mesenteric artery
TA: Takayasu arteritis
TNF-α: Tumour necrosis factor alpha
USS: Ultrasound
VDI: Vasculitis Damage Index
