# Supplementary material for: Takayasu arteritis in childhood: retrospective experience from a tertiary referral centre in the United Kingdom
Source: Arthritis Res Ther. 2015 Feb 25;17(1):36. doi: 10.1186/s13075-015-0545-1 (PMC4392477; doi:10.1186/s13075-015-0545-1)
Supplement: Additional file 1: — Disease Extent Index for Takayasu Arteritis tool. [file 13075_2015_545_MOESM1_ESM.pdf]

Front Page for Disease Extent Index for Takayasu Arteritis - DEI.Tak

1. Investigator: Assessment date:
2. Medical Centre:
3. Centre Code: Patient #:
4. Patient Name: Age: Sex:

   Address

   Postal code contact telephone

5. Place of birth: Parents birthplace Father: Mother:
6. Year of disease onset: Presenting symptom(s):
7. Year of diagnosis: Angio performed? Date?
8. Other imaging (specify, with dates):
9. Treatment
   - Current drugs
   - Past drugs
   - Surgery
10. Physician's Global Opinion (**PGO)** score of **current** disease activity:

    A - active disease **B - g**rumbling or persistent disease **C** - inactive disease
11. Name + Signature of doctor completing assessment

Draft 3 – Mar'06

DEI.Tak – Disease Extent Index for Takayasu's Arteritis

Patient name:

Tick Box only if abnormality is present (new or worse within 6/12), with duration for each symptom.
Tick box only if abnormality is attributed to current vasculitis.

Visit Date :
Investigator:

	PRESENT	duration
**1. SYSTEMIC**		
None	☐	
Malaise/Wt. Loss>2Kg	○	
Myalgia/Arthralgia/Arthritis.	○	
Headache	○	
Fever	○	
**2. CUTANEOUS**		
None	☐	
Gangrene	○	
Other Skin Vasculitis	○	
**3. MUCOUS MEMBRANES**		
none	☐	
Present	○	
**4. EYES**		
None	☐	
Blurred Vision	○	
Sudden Vision Loss	○	
Other	○	
**5. ENT**		
None	☐	
Present	○	
**6. CHEST**		
None	☐	
Persistent Cough	○	
Dyspnea/Wheeze	○	
Hemoptysis/Hemorrhage	○	
Massive Hemoptysis	○	
Respiratory Failure	○	

Chest Radiology ○
Active Vasculitis confirmed ○

	PRESENT	duration
**8. ABDOMEN**		
None	☐	
Severe Abdominal Pain	○	
Bloody Diarrhea	○	
Gut Perforation/Infarct	○	

Surgical Opinion / tests ○
Active Vasculitis confirmed ○

	PRESENT	duration
**9. RENAL**	☐	
None		
Hypertension (Diastole >90)	○	
"" Systolic >140	○	
Proteinuria (>1+/0.2g/24H)	○	
Hematuria (>1+/10RBC/ml)	○	
Creatinine (125-249 μmol/L)	○	
Creatinine (250-499 μmol/L)	○	
Creatinine (>500 μmol/L)	○	
Rise in creatinine >30% or > 25% fall in creatinine clearance.	○	
**10. Nervous System**		
None	☐	
Organic Confusion/Dementia	○	
Seizures (not hypertensive)	○	
Stroke	○	
Syncope	○	
Cord Lesion	○	
**11. Genitourinary System**	☐	
None		
Sexual Impotence	○	
Abortions	○	

**7. CARDIOVASCULAR SYSTEM**	
none	☐
Bruits (see 7a)	○
Pulse Inequality (See 7b)	○
Pulse Loss (See 7c)	○
Pulse Loss with threatened loss of limb.	○
Claudication (See7d)	○
Carotidodynia	○
Aortic Incompetence	○
Pericardial Pain/Rub	○
Ischemic Cardiac Pain	○
Congestive Cardiac Failure	○

Cardiology Opinion/Tests

Active Vasculitis confirmed	○
Pericarditis	○
Myocardial Infarct/Angina	○
Cardiomyopathy	○

**7a. Bruits**	**R**	**L**
Carotid	○	○
Vertebral	○	○
Subclavian	○	○
Renal	○	○
Abdominal	○	
Inguinal	○	○

7b. Pulse and BP Inequality
Present ○

**7c. Pulse Loss**		
Carotid	○	○
Subclavian	○	○
Brachial	○	○
Radial	○	○
Femoral	○	○
Popliteal	○	○
Posterior Tibial	○	○
Dorsalis Pedis	○	○

**7d. Claudication**	
Arm	○
Leg	○
Neck	○

12. Other Vasc items:

ESR **CRP**

13. PGO (Active / Grumbling or persistent / Inactive):

Revised form by M.R Sivakumar, R.Misra, and P.A.Bacon – Dec 2005

Disease Extent Index for Takayasu Arterits (DEI.Tak)

M.R.Sivakumar, R.N.Misra and P.A.Bacon (March 2006)

An Introduction and Glossary of Terms

Purpose of assessment

Standard therapy for systemic vasculitis has markedly improved the acute mortality but relapse remains a problem. Mortality is no longer an acceptable end point for studies. Serial studies require detailed assessment of disease status in order to estimate the degree of improvement achieved together with any accumulated scars or damage. Both contribute to the long term outcome and are needed for proper comparison of new regimes. In this document, we describe the use of a Disease Extent Index for Takayasu Arteritis (DEI.Tak).

Attribution.

DEI.Tak is designed to document those features which are directly due to Takayasu Arteritis. These items were derived by consensus opinion from experts in the clinical management of Takayasu Arteritis in India and in the U.K.
No attempt is made to distinguish clinical features which represent very new or worsening disease activity from those which represent smouldering disease activity. Thus all features attributable to aorto-arteritis activity within the past 6 months are recorded. However it is very important not to confuse smouldering disease activity with persistent damage, where there is no current disease activity. Damage is defined as the presence of non-healing scars and does not give any indication of current disease activity. As Takayasu is a slowly progressive disease, all features that have been present without any progression for longer than 6 months are classed as damage and are not included in the DEI.Tak.
DEI.Tak is designed to record features which are attributable to Takayasu Arteritis, after exclusion of other obvious causes such as infection, hypertension, etc. You are asked to record only those abnormalities which you can attribute directly to Takayasu Arteritis. A glossary of terms used is attached to aid standardisation of usage. New users are particularly directed to this section to aid learning the approach to this instrument.

Recording Disease Extent Index for Takayasu Arteritis

It would be most efficient if you could record the DEI.Tak for Takayasu Arteritis whilst seeing the patient. **Please fill in the front sheet on each patient, then go through the assessment of disease extent.** The DEI.Tak can be used as checklist of items that you would normally wish to assess in the clinic. The list is a combination of clinical signs and symptoms, as well as information obtained from additional tests (e.g., chest x-rays) or subspecialty consultations.

New Patients

If the patient is being evaluated for the first time and has not been treated, all of the abnormalities noted should be recorded. After going through the entire list of items, remember to consider adding any other significant items directly relevant to Takayasu to the “Other” section. This section is to ensure that the new DEI.Tak is comprehensive.

Follow-up Patients

If the patient is being evaluated in follow-up, there may be some abnormalities that are new or worse within the previous 6 months. Record these and note the duration (in months). Other abnormalities that are still present despite treatment, but are neither new nor worse in the past 6 months, count as damage and are not included in this DEI.Tak assessment.

Checking the Boxes

Check one of the ⬭ boxes for each item only if you ascribe the abnormality to the presence of TA. If no abnormalities attributable to TA are present in a given organ system, check the □ “none” box . In this way, we can be certain that you did not overlook an organ system on the scoring sheet. Sometimes you will have patients in whom abnormalities are present that are not due to TA (e.g., hematuria due to urinary infection or cyclophosphamide toxicity). In these cases, you should NOT record them in the DEI.Tak list, even though they are present, because they are not caused by TA.

Necessity for “Judgement Calls”

As in clinical practice, one must sometimes make “judgement calls” in scoring DEI.Tak For example, fresh loss of pulsations may be a symptom to intensify treatment. As a general rule, a symptom or sign that would lead you to consider altering the therapy directed at control of the Takayasu is one that you attribute to the disease and include when scoring the DEI.Tak.

Calculating the DEI.Tak Score

The DEI.Tak score is calculated by adding all of the positive boxes ⬭ marked (but not of course the none boxes □). The individual items are not weighted – but serious items in 7, the CVS, such as pulse loss and bruit lead to further boxes in 7 a, b, c, or d, to delineate the site. These boxes ⬭ when marked are also included in the total, so that the overall score is effectively biased toward major items.

Physician’s Global Assessment - PGO.

Finally, record your assessment of the current overall disease activity in this case in one of three categories - **A** - active disease; **B - g**rumbling or persistent disease; or **C** - inactive disease. Remember that you should not be influenced by the presence of any accumulated damage, complication of treatment, social/emotional problems, or other issues not related to TA, when forming a global opinion of disease activity.

Disease Extent Index for Takayasu Arteritis

GLOSSARY OF TERMS

ATTRIBUTION: disease features are scored only when they are attributable to active vasculitis, after exclusion of other obvious causes (infection, hypertension, etc.).

DURATION: items are added only when newly present or worse within the past 6 months.

It is essential to apply these principles to <u>each</u> item below:

Glossary definitions used in DEI.Tak

Remember that in most instances, you will be able to complete the whole record when you see the patient. However, for some features, further information (from specialist opinion or further tests) is required before entry. We would suggest that you leave these items blank, and once the information is available, please remember to take the time to fill in the information.

**1. Systemic**	
**Arthralgia:**	pain in the joints.
**Arthritis:**	joint inflammation
**Fever:**	Documented temperature elevation. The value refers to oral/axillary temperatures. Rectal temperatures are 0.5^0C higher
**Headache:**	Pain in the head

**2. Cutaneous**	
**Gangrene:**	Extensive tissue necrosis (e.g. digit)
**Other skin Vasculitis**	Purpura**:** Petechiae (small red spots), palpable purpura, or ecchymoses (large plaques) in skin; nailfold or nail-edge infarcts

**3. Mucous Membranes**	This system is rarely involved in Takayasu and no items are regarded as specific to this disease. If any mucous membrane involvement occurs that you attribe to Takayasu, tick the “Present” box and write in the detail.

4. Eyes

**Sudden visual loss:**	Sudden loss of vision requiring ophthalmological assessment.
**Blurred vision:**	Haziness in eyesight

**5. ENT**	This system is rarely involved in Takayasu and no items are regarded as specific to this disease. If any ENT involvement occurs that you attribute to Takayasu, tick the “Present” box and write in the detail.

**6. Chest**	
**Persistent Cough:**	Continuous cough
**Dyspnoea/Wheeze:**	Difficulty in breathing or shortness of breath
**Haemoptysis/**<br><br>**Haemorrhage**	Production of blood stained sputum. Other causes (e.g. infection, cancer) should be excluded<br><br>Shifting pulmonary infiltrates, often with a “bats wing” pattern and associated with fall in haemoglobin levels.<br>Other causes of bleeding should be excluded
**Massive haemoptysis**	Major pulmonary bleeding with plentiful blood stained sputum or frank blood. usually associated with signs of shock
**Respiratory Failure:**	Incapacitating persistent dyspnoea, which may require oxygen.

**7. Cardiovascular System**	
**Bruits :**	Audible to and fro sound over arteries by auscultation with a stethoscope. Tick box and then move to 7a. to delineate site(s) involved. Check all of these - Carotid; Vertebral; Subclavian; Renal; Abdominal; Inguinal
**Pulse Inequality**	Feeble pulse on one side when compared to a similar pulse on the opposite side. Move to 7b. and check for difference in systolic pressure > 10 mmHg between the two limbs
**Pulses Loss**	Loss of previously felt pulse under observation.<br>Tick box and then move to 7c. to record anatomic site(s) involved. Check all of Carotid; Subclavian; Brachial; Radial; Femoral; Popliteal; Posterior tibial; Dorsalis pedis
**Pulse Loss with threatened limb loss**	Loss of previously felt pulse with present or impending gangrenous changes
**Claudication**	Pain during movements or activity. Tick box and move to 7d. to record site in arm or leg. Exercise-related neck pain or subclavian steel may also be recorded here as claudication
**Carotidodynia**	Tenderness or pain during palpation of the Carotid arteries
**Aortic Incompetence**	Leakage of the Aortic valve detected clinically or by ECHO Cardiography

**7. CVS continued**	
**Pericardial Pain/Rub**	Anterior chest pain relieved by sitting up; high pitched scratching noise audible over the left precordium during any part of the cardiac cycle by auscultation
**Ischemic Cardiac pain**	Chest pain during exertion, relieved by rest or trinitrin
**Congestive cardiac failure**	Fluid retention with swelling in the feet/body, associated with basal lung crepitations and elevated JVP due to pump failure

**8. Abdominal**	All items here require surgical opinion/tests to confirm active vasculitis
**Severe abdominal pain:**	persistent intense pain without other clear cause and attributed to vasculitis.
**Bloody diarrhoea:**	overt blood-stained stools, not due to known inflammatory bowel disease,.
**Gut perforation/ infarction:**	typical pain and peritonism includes gall bladder or appendix. *Confirmed by X-ray or at surgery.*

**9. Renal**	
**Hypertension:**	Elevated B.P., diastolic (> 90 mmHg) and or systolic(>140mm/Hg)
**Proteinuria:**	Albuminuria of more than 1+ on dipstick or > 0.2g in a 24 hour collection
**Haematuria:**	≥1+ on dipstick urinalysis; ≥10 rbc/ml, or red cell casts seen on urine microscopy. Infection should be excluded.
**Creatinine (125-249 μmol/L)**	Serum levels by standard lab analysis
**Creatinine (250-499 μmol/L)**	Serum levels by standard lab analysis
**Creatinine (>500 μmol/L)**	Serum levels by standard lab analysis
***Rise in creatinine > 30% or creatinine clearance fall > 25%:**	Significant deterioration in renal function attributable to active vasculitis.

**10. Nervous System**	
**Organic confusion/ Dementia**	Overt disorientation, loss of memory or prolonged mental reaction time
**Seizures (not hypertensive):**	Paroxysmal electrical discharges in the brain and producing characteristic physical changes including tonic and clonic movements and certain behavioural changes.
**Stroke:**	Cerebrovascular accident resulting in focal neurological signs such as paresis, weakness, etc.
**Syncope:**	Reduced B.P. and cerebral perfusion, causing loss consciousness.
**Cord lesion:**	Transverse myelitis with lower extremity weakness or sensory loss with loss of sphincter control (rectal and urinary bladder).

**11. Genitourinary System**	
**Sexual impotence:**	Inability to obtain and maintain satisfactory erection or premature ejaculation.
**Abortions:**	Spontaneous foetal miscarriages during pregnancy

**12. Other vasculitis items**	Any item attributable to active aorto-arteritis which is not included above may be recorded here if it is new or worse within the past 6 months
**Non-specific lab measures of inflammation**	
**ESR:**	Measured by Westergren's method, it is a broad assay of acute phase reactants but varies between labs and stays elevated after acute inflammation
**CRP:**	The best test for an acute phase reactant and a sensitive indicator of current active inflammation.

**13. PGO:**	Physicians assessment of the overall status of the current disease activity in this patient Please circle or underline one of three categories - **A** Active; **B** Grumbling or persistent; **C** Inactive.
