# Supplementary material for: Takayasu arteritis in childhood: retrospective experience from a tertiary referral centre in the United Kingdom
Source: Arthritis Res Ther. 2015 Feb 25;17(1):36. doi: 10.1186/s13075-015-0545-1 (PMC4392477; doi:10.1186/s13075-015-0545-1)
Supplement: Additional file 2: — Indian Takayasu Arteritis Activity Score tool. [file 13075_2015_545_MOESM2_ESM.doc]

| ITAS2010 – IndianTakayasu’s Arteritis Activity Score | |
| --- | --- |
| Tick Box only if abnormality is present and new or worse within the past 3/12.  Tick box only if abnormality is ascribed to current, active vasculitis. | Name:  Unit Number: Visit Date:  Investigator: |

**PRESENT**

**1. SYSTEMIC**

None

Malaise/Wt. Loss>2Kg

Myalgia/Arthralgia/Arthritis.

Headache

**2. ABDOMEN**

None

Severe Abdominal Pain

**3. Genitourinary System**

None

Abortions

**6. CARDIOVASCULAR SYSTEM**

none

**Bruits (see 6a)**

**Pulse Inequality (See 6 b)**

**New Loss of Pulses (See 6c)**

**Claudication (See 6d)**

**Carotidodynia**

Aortic Incompetence

Myocardial Infarct/Angina

Cardiomyopathy/cardiac failure

**Other Vasculitis items:**

**ESR CRP**

**Item scores = 0 = 1 = 2**

**Scoring ITAS2010 :** Add all scores. In CVS , if both boxed circle and circle are ticked, add both (see glossary) **.**

**Scoring ITAS.A** including acute phase response

- for ESR, score ITAS plus: 0 for <20; 1 for ESR 21-39;

2 for ESR 40- 59; and 3 for >60 mm ESR /hr

#### - for CRP score ITAS plus: 0 for CRP <5; 1 for CRP 6-10;

#### 2 for CRP 11-20; and 3 for >20 mg/dl

####

**PRESENT**

**4. RENAL**

None

**Hypertension (Diastole >90)**

“” Systolic >140

**5. Nervous System**

None

**Stroke**

Seizures (not hypertensive)

Syncope

Vertigo/dizzyness

**6a. Bruits R L**

Carotid

Subclavian

Renal

**6b. Pulse and BP Inequality**

Present

**6c. Pulse Loss**

Carotid

Subclavian

Brachial

Radial

Femoral

Popliteal

Posterior Tibial

Dorsalis Pedis

**6d. Claudication**

Arm

Leg

**Physician Global Assessment**

**Active / Grumbling or persistent / Inactive**

**New Imaging Y / N? If Y - specify ___________________**

#### ITAS2010 form. M.R Sivakumar, R.Misra, D.Danda & P.A.Bacon - Mar’10

**ITAS.A form – ibid Oct 2012**
