# Supplementary material for: Takayasu arteritis in childhood: retrospective experience from a tertiary referral centre in the United Kingdom
Source: Arthritis Res Ther. 2015 Feb 25;17(1):36. doi: 10.1186/s13075-015-0545-1 (PMC4392477; doi:10.1186/s13075-015-0545-1)
Supplement: Additional file 3: — Indian Takayasu Arteritis Activity Score tool glossary. [file 13075_2015_545_MOESM3_ESM.docx]

Supplementary Glossary for terms used in the ITAS2010 and ITAS.A

Purpose of assessment and scoring of ITAS.

The purpose of ITAS2010 is to capture manifestations related to TA that is attributable to new disease activity in the last 3 months **Disease features are only scored when they are attributable to vasculitis, after excluding other causes such as infection. The ITAS2010 captures clinical activity, acute phase response and image abnormalities, though in general imaging modalities are done less frequently and not at 3 months. While scoring CVS features such as pulse loss, any image done prior to analysis should be taken into account.**

ITAS2010 thus differs from DEI.Tak (the Disease Extent Index for Takayasu) which aims to include all features of the disease. Any features not included which the physician considers as attributable to TA, due to current activity of disease or requiring immunosuppressive treatment may be recorded in the “Other Vasculitis items” box. Persistent features present >3/12, which are considered to be the end result of inflammation, complications of treatment and are not amenable to reverse with medical treatment, should be excluded.

Instruction for completing the form: Fill in patients Name and Unit number (or hospital/Clinic ID details). The form must then be signed (name and signature).

GLOSSARY OF TERMS

1. Systemic

Malaise : a state of not feeling well or easy fatigability. In TA it may be due to systemic inflammation.

Weight loss: unexplained loss of > 2Kg. Exclude intentional slimming and other conditions such as diabetes mellitus, tuberculosis, HIV, malignancy etc.

Myalgia: generalized muscle aches and pains, due to systemic inflammation. Not to be confused with claudication pain which is worse on physical activity of a selected group of muscles .

Arthralgia: Joint pains

Arthritis: Joint inflammation with swelling and tenderness, often with associated skin erythema and warmth, with or without restriction of movement

Fever: Documented temperature ≥ 38^0^ C

Headache: new or unusual severe persistent headache. In TA it is usually associated with hypertension

2. Abdomen

Severe abdominal pain: Pain presenting as an emergency with features of bowel ischemia, best confirmed by imaging or surgery, with exclusion of other conditions giving rise to severe abdominal pain such as peptic ulcer disease, hepatic or gall bladder disease, pancreatitis, stones in gall bladder or kidneys or urinary system, peritoneal inflammation , appendicitis, colitis, polyps.

3. Genito-urinary

Abortions: spontaneous foetal loss within 12 weeks, not ascribed to genetics, maternal infections, antibodies such as lupus anticoagulant or any other maternal conditions.

4. Renal

**Hypertension:** Diastolic BP > 90 or systolic BP>140 measured at the time of examination. If the patient has been observed by a physician to be hypertensive for the first time within 3 months and is on antihypertensive treatment, new hypertension should be scored even if the BP is now controlled.

5 . Neurological

Stroke: cerebro-vascular accident producing focal neurological signs. Other causes such as atherosclerosis should be considered and neurological advice sought if suspected.

Siezures: characteristic clonal and tonic movements often associated with behavioural change, due to paroxysmal electrical discharges in the brain.

Syncope: sudden temporary loss of consciousness in the absence of trauma, intoxication etc, often associated with unexplained falls.

Vertigo/dizziness: spells of unstable balance, difficulty in standing steadily or sensations of spinning without external causes.

6. Cardiovascular system. CVS bears the brunt of the disease in TA and five items are weighted to score 2. Four of these lead the observer on to document the extent of involvement. All the potential sites need examining at each assessment and images such as CT or MR Angio done prior to assessment should be taken into account for featuring pulse loss.

Bruits: Audible to and fro sounds over arteries. If present tick box at 6 and also document which arteries are involved in appropriate box at 6a. It is essential to auscultate carotid, subclavian and renal arteries bilaterally.

Pulse and BP Inequality: Feeble pulse on one side as compared to the same pulse on the opposite limb. Confirm by BP measurement and if systolic pressure differs by > 10 mmHg between the 2 limbs tick 6b.

New Pulse Loss: Absence of previously observed pulse. Tick the square and then record the specific vessel losses in 6c.

Claudication: Pain in limb muscles during movements or activity. Tick box and move to 6d to record site of claudication in the arm or leg. Exercise-related subclavian steel symptoms may also be recorded here as claudication.

Carotidodynia: Tenderness or pain during palpation of the Carotid arteries

Aortic Incompetence: Leakage of the Aortic valve detected clinically or by ECHO cardiography.

Ischemic Cardiac pain: Precordial angina pain precipitated by meals or exertion, relieved by rest or nitrates

Mycardial infarction: Typical clinical picture of severe chest/arm pain supported by ECG and enzyme changes. Documentation by cardiologist or physicians is important.

7. Other vasculitis items

Any item which is not included above but is considered by the physician as due to active disease in the case of TA being examined.

8. Non-specific lab measures of inflammation

ESR: Measured by Westergren method,

CRP: Conventional C reactive protein as measured by a quantitative laboratory assay.

9. PGO: Physicians assessment of the overall status of the current disease activity in this patient. Circle or underline one of three categories - **A** current active disease;

**B** Grumbling or persistent disease; **C** Inactive case.

10. New Imaging. If done in the last 3 months, document any change from prior imaging particularly vessel narrowing or blockage plus any changes in vessel

Calculation of ITAS.2010**:** Add all scores. Note that 5 items in the CVS, together with diastolic Hypertension and Stroke are weighted to score 2. All other items score 1 only. In CVS, if both boxed circle and circle(s) are ticked, add both**.** For example, a patient new with a loss of pulse" (2) observed in right carotid (1), right subclavian (1) and left brachial (1) arteries will have a score of 5 for this section.

**Calculation of ITAS.A -** calculate all clinical scores for ITAS2010 and add score for acute phase assay as follows:

**ITAS -ESR** - add 0 for ESR <20, 1 for ESR 21-39; 2 for ESR 40-59; and 3 for ESR >60 mm.

nb ESR /hr by Westergren method.

#### ITAS-CRP – add 0 for CRP<5 ; 1 for CRP 6-1; for CRP 11-20: and 3 for CRP >20 mg/dl
