# Supplementary material for: Takayasu arteritis in childhood: retrospective experience from a tertiary referral centre in the United Kingdom
Source: Arthritis Res Ther. 2015 Feb 25;17(1):36. doi: 10.1186/s13075-015-0545-1 (PMC4392477; doi:10.1186/s13075-015-0545-1)
Supplement: Additional file 4: — Paediatric Vasculitis Activity Score tool. [file 13075_2015_545_MOESM4_ESM.pdf]

PAEDIATRIC VASCULITIS ACTIVITY SCORE

○ Tick "**Active**" box **only** if abnormality due to active vasculitis is newly present or worse over the last 4 weeks or persists for less than 3 months. After that, if ALL items are persistent and represent smouldering/low grade/grumbling disease, and there are no new/worse features, please tick the box at the bottom right corner. At the very first assessment all active items are considered as active/worse. If there are no abnormalities in a system, please tick the "**None**" box. For items present longer than 3 months refer to the Vasculitis Damage Index to score damage.

	None	Active
**1. General**	○	
Myalgia		○
Arthralgia or arthritis		○
Fever ≥ 38.0 ^{0}C		○
Weight Loss ≥ 5% body weight		○
**2. Cutaneous**	○	
Polymorphous exanthema		○
Livedo		○
Panniculitis		○
Purpura		○
Skin nodules		○
Infarct (nail edge lesion, splinter haemorrhage)		○
Ulcer (full-thickness necrosis)		○
Gangrene (extensive necrosis)		○
Other skin vasculitis (specify below)		○
**3. Mucous membranes/eyes**	○	
Mouth ulcers/granulomata		○
Genital ulcers		○
Adnexal inflammation		○
Significant proptosis		○
Red eye (Epi)scleritis		○
Red eye conjunctivitis/ blepharitis/keratitis		○
Uveitis		○
Blurred vision		○
Sudden visual loss		○
Retinal vasculitis/retinal vessel thrombosis/ retinal exudates/haemorrhages		○
**4. ENT**	○	
Nasal discharge/crusts/ulcers/granuloma		○
Paranasal sinus involvement		○
Subglottic stenosis/ hoarseness /stridor		○
Conductive hearing loss		○
Sensorineural hearing loss		○
**5. Chest**	○	
Wheeze or expiratory dyspnea		○
Endobronchial/endotracheal involvement		○
Nodules or cavities		○
Pleural effusion/pleurisy		○
Infiltrate		○
Massive haemoptysis/Alveolar haemorrhage		○
Respiratory failure		○

	None	Active
**6. Cardiovascular**	○	
Loss of pulses		○
Bruits over accessible arteries		○
Blood pressure discrepancy		○
Caudication of extremities		○
Ischaemic cardiac pain		○
Cardiomyopathy		○
Congestive cardiac failure		○
Valvular heart disease		○
Pericarditis		○
**7. Abdominal**	○	
Abdominal pain		○
Peritonitis		○
Blood in stools or bloody diarrhoea		○
Bowel ischaemia		○
**8. Renal**	○	
Hypertension >95th centile (for height)		○
Proteinuria >0.3 g/24h,>20mmol/mg creatinin		○
Haematuria ≥2+ or 5 rbc/hpf or red cell casts		○
GFR 50-80ml/min/1.73 m^2		○
GFR 15-49 ml/min/1.73 m^2		○
GFR <15 ml/min/1.73m^2		○
Rise in creatinine > 10% or Creatinine clearance (GFR) fall > 25%		○
**9. Nervous system**	○	
Headache		○
Meningitis/encephalitis		○
Organic confusion/cognitive dysfunction		○
Seizures (not hypertensive)		○
Stroke		○
Cord lesion		○
Cranial nerve palsy		○
Sensory peripheral neuropathy		○
Motor mononeuritis multiplex		○
**10. OTHER**	○	
		○
		○
**NO NEW/WORSE DISEASE** :		
Tick here if there is no new/worse abnormality present in ANY of the systems above and active items represent low grade grumbling disease		

PVAS 2008 *17/09/2008 P.Dolezalova Version 1.0*

Modified from Luqmani R.A., Bacon P.A., Moots R.J. et al (1994) BVAS in systemic necrotizing vasculitis. Quart J Med 87: 671-8
