# Supplementary material for: Takayasu arteritis in childhood: retrospective experience from a tertiary referral centre in the United Kingdom
Source: Arthritis Res Ther. 2015 Feb 25;17(1):36. doi: 10.1186/s13075-015-0545-1 (PMC4392477; doi:10.1186/s13075-015-0545-1)
Supplement: Additional file 5: — Paediatric Vasculitis Activity Score tool glossary. [file 13075_2015_545_MOESM5_ESM.pdf]

**Glossary and scoring for PVAS.** GENERAL RULE: disease features are scored only when they are due to active vasculitis, after excluding other causes (e.g. infection, hypertension, etc.). If the feature is due to active disease, it is scored in the boxes. It is essential to apply these principles to each item below. Scores have been weighted according to the severity which each symptom or sign is thought to represent. Tick "Persistent Disease" box if all the abnormalities are due to active (but not new or worse) vasculitis. If any of the abnormalities are due to new/worse disease , DO NOT tick the "Persistent Disease" box. For some features, further information (from specialist opinion or further tests) is required if abnormality is newly present or worse. Remember that in most instances, you will be able to complete the whole record when you see the patient. However, you may need further information before entering some items. Please leave these items blank, until the information is available, and then fill them in. For example, if the patient has new onset of stridor, you would usually ask an ENT colleague to investigate this further to determine whether or not it is due to active Wegener's granulomatosis.		**PVAS persistent**	**PVAS new/ worse**
**1. General**	**Maximum scores**	**2**	**3**
Myalgia	Diffuse, spontaneous, hard to localize muscle pain or tenderness on muscle palpatıon. Exclude fibromyalgia.	1	1
Arthralgia or arthritis	Joint pain in any number of joints or presence of objective signs of active synovitis: intraarticular swelling due to synovial proliferation and/or joint effusion with limited range of movement and/or pain on movement or joint tenderness. Any number of joints.	1	1
Fever ≥ 38.0 ^{0}C	Documented temperature elevation >38ºC. The value refers to axillary/oral temperature (rectal temperature 0.5 ºC higher). Exclude infections by appropriate cultures, serology and PCR methods.	2	2
Weight Loss ≥ 5% body weight	At least 5% loss of body weight (not fluid) having occurred since last assessment or in the 4 weeks not as a consequence of dieting	2	2
**2. Cutaneous**	**Maximum scores**	**3**	**6**
Polymorphous exanthema	Non-haemorrhagic, non-necrotising skin eruption of any type or combined types. Exclude allergy/drug reaction/infection	1	1
Livedo	Purplish reticular pattern usually irregularly distributed around subcutaneous fat lobules, often more prominent with cooling, common over foot margins. Exclude antiphospholipid syndrome.	1	1
Panniculitis	Single or multiple tender deep subcutaneous nodules caused by inflammation of deep subcutaneous tissue with typical histopathology findings if biopsy performed	1	1
Purpura	Petechiae (small red spots), palpable purpura, or ecchymoses (large plaques) in skin or oozing (in the absence of trauma) in the mucous membranes.	1	2
Skin nodules	Subcutaneous nodules, often along arteries, tender on palpation.	1	1
Infarct	Nail edge lesion, splinter haemorrhage or flea bite lesion of small vessel vasculitis	1	1
Ulcer	Area of full-thickness skin/subcutaneous tissue ulceration/necrosis	1	4
Gangrene	Extensive skin/subcutaneous tissue/underlying structure necrosis, digital phalanx or other peripheral (nose, ear tips) necrosis/gangrene	2	6
Other skin vasculıtıs	Vasculıtıs dıfferent from prevıous e.g. subcutaneous swelling/oedema due to capillary leak in small vessel involvement, Raynaud´s phenomenon etc.	1	1
**3. Mucous membranes/eyes**	**Maximum scores**	**3**	**6**
Mouth ulcers/granulomata	Aphtous stomatitis, ischaemic ulcers and/or granulomatous inflammation in oral cavity. Exclude other causes (SLE, infection)	1	2
Genital ulcers	Ulcers localised in the genitalia or perineum, excluding infections.	1	1
Adnexal inflammation	Salivary (diffuse, tender swelling unrelated to meals) or lacrimal gland inflammation. Exclude other causes (infection). Specialist opinion preferably required.	2	4
Significant proptosis	Protrusion of the eyeball due to significant amounts of inflammatory in the orbit; if unilateral, there should be a difference of 2 mm between one eye and the other. This may be associated with diplopia due to infiltration of extra-ocular muscles. Developing myopia (measured on best visual acuity, see later) can also be a manifestation of proptosis	2	4
Red eye (Epi)scleritis	Inflammation of the sclerae (specialist opinion usually required). Can be heralded by photophobia.	1	2
Red eye conjunctivitis<br><br><br>Blepharitis<br><br>Keratitis	Inflammation of the conjuctivae (exclude infectious causes and excluding uveitis as cause of red eye, also exclude conjunctivitis sicca which should not be scored as this is not a feature of active vasculitis); (specialist opinion not usually required).<br>Inflammation of eyelids. Exclude other causes (trauma, infection). Usually no specialist opinion is required<br>Inflammation of central or peripheral cornea as evaluated by specialist	1	1
Blurred vision	Altered measurement of best visual acuity from previous or baseline, requiring specialist opinion for further evaluation.	2	3
Sudden visual loss	Sudden loss of vision requiring ophthalmological assessment.		6
Uveitis	Inflammation of the uvea (iris, ciliary body, choroid) confirmed by ophthalmologist.	2	6
Retinal vasculitis	Retinal vessel sheathing on examination by specialist or confirmed by retinal fluoroscein angiography	2	6
Retinal vessel thrombosis	Arterial or venous retinal blood vessel occlusion		
Retinal exudates	Any area of soft retinal exudates (exclude hard exudates) seen on ophthalmoscopic examination.		
Retinal haemorrhages	Any area of retinal haemorrhage seen on ophthalmoscopic examination.		
**4. ENT**	**Maximum scores**	**3**	**6**
Bloody nasal discharge/ nasal crusts/ulcers and/or granulomata	Bloody, mucopurulent, nasal secretion, light or dark brown crusts frequently obstructing the nose, nasal ulcers and/or granulomatous lesions observed by rhinoscopy	2	4
Paranasal sinus involvement	Tenderness or pain over paranasal sinuses usually with pathologic imaging (CT, MR, x-ray, ultrasound)	1	2
Subglottic stenosis	Stridor and hoarseness due to inflammation and narrowing of the subglottic area observed by laryngoscopy	3	6
		**PVAS persistent**	**PVAS new/worse**
Conductive hearing loss	Hearing loss due to middle ear involvement confirmed by otoscopy and/or tuning fork examination and/or audiometry	1	3

Sensorineural hearing loss	Hearing loss due to auditory nerve or cochlear damage confirmed by audiometry	2	6
**5. Chest**	**Maximum scores**	**3**	**6**
Wheeze or expiratory dyspnea	Clinical signs of bronchial obstruction on examination	1	2
Nodules or cavities	New lesions, detected by CXR		3
Pleural effusion/pleurisy	Pleural pain and/or friction rub on clinical assessment or new onset of radiologically confirmed pleural effusion. Other causes (e.g. infection, malignancy) should be excluded	2	4
Infiltrate	Detected by CXR or CT scan. Other causes (infection) should be excluded	2	4
Endobronchial involvement	Endobronchial pseudotumor or ulcerative lesions. Other causes such as infection or malignancy should be excluded. NB: smooth stenotic lesions to be included in VDI; subglottic lesions to be recorded in the ENT section.	2	4
Massive haemoptysis/alveolar haemorrhage	Major pulmonary bleeding, with shifting pulmonary infiltrates; other causes of bleeding should be excluded if possible	4	6
Respiratory failure	Dyspnoea which is sufficiently severe as to require artificial ventilation	4	6
**6. Cardiovascular**	**Maximum scores**	**3**	**6**
Loss of pulses	Loss of pulses in any vessel detected clinically; this may include loss of pulses leading to threatened loss of limb	1	4
Bruits over accessible arteries	Audible murmurs on auscultation or palpable bruits/thrills over large arteries and aorta	1	2
Blood pressure discrepancy	>10 mm Hg difference in any limb	1	2
Claudication of extremities	Focal muscle pain elicited usually by physical activity	1	2
Ischaemic cardiac pain	Typical clinical history of cardiac pain leading to myocardial infarction or angina.	2	4
Cardiomyopathy	Significant impairment of cardiac function due to poor ventricular wall motion confirmed on echocardiography.	3	6
Congestive cardiac failure	Heart failure by history or clinical examination	3	6
Valvular heart disease	Significant valve abnormalities in the aortic mitral or pulmonary valves detected clinically or echocardiographically.	2	4
Pericarditis	Pericardial pain &/or friction rub on clinical assessment	1	3
**7. Abdominal**	**Maximum scores**	**5**	**9**
Abdominal pain	Persistent or recurrent abdominal pain, other than vasculitic causes excluded	2	4
Peritonitis	Acute abdominal pain with peritonism/peritonitis due to perforation/infarction of small bowel, appendix or gallbladder etc., or acute pancreatitis confirmed by radiology/surgery/elevated amylase	3	9
Blood in stools or bloody diarrhoea	Overt or occult blood in stools or bloody diarrhoea of recent onset; inflammatory bowel disease, anal fissure and infectious causes excluded.	2	6
Bowel ischaemia	Severe and recurrent abdominal pain often with GI bleeding due to ischaemic necrosis of the gut confirmed by imaging or at surgery, with typical appearances of aneurysms or abnormal vasculature characteristic of mesenteric vasculitis.	3	9
**8. Renal**	**Maximum scores**	**6**	**12**
Hypertension >95th centile	Systolic blood pressure greater than 95th centile by age and hight	1	4
Proteinuria >0.3g/24hr or >20mmol/mg Cr	Persistent >20 mmol/mg creatinine and/or >0.3 g/24 hours.	2	4
Haematuria ≥5 rbc/hpf or red cell casts	10 or more RBC per hpf (high power field), excluding urinary infection and urinary lithiasis (stone)	3	6
GFR 50-80ml/min/1.73 m2	Calculated or measured GFR 50-80mls/min/1.73m2.	2	4
GFR 15-49 ml/min/1.73 m2	Calculated or measured GFR 15-49mls/min/1.73m2.	3	6
GFR <15 mk/min/1.73m2	Calculated or measured GFR <15 mls/min/1.73m2	4	8
Rise in creatinine > 10% or Creatinine clearance (GFR) fall > 25%	Significant deterioration in renal function attributable to active vasculitis. Rise in creatinine >10% when compared to previous value or fall in calculated or measured GFR >25%		6
**9. Nervous system**	**Maximum scores**	**6**	**9**
Headache	New, unaccustomed & persistent headache	1	1
Meningitis/encephalitis	Severe headache with neck stiffness ascribed to inflammatory meningitis after excluding infection/bleeding	1	3
Organic confusion/cognitive dysfunction	Impaired orientation, memory or other intellectual function in the absence of metabolic, psychiatric, pharmacological or toxic causes.	1	3
Seizures (not hypertensive)	Focal motor, generalised or psychomotoric epileptic paroxysm, due to CNS vasculitis. Exclude idiopathic epilepsy, febrile seizures	3	9
Stroke	Cerebrovascular accident resulting in focal neurological signs as paresis, weakness etc.	3	9
Cord lesion	Transverse myelitis with lower extremity weakness or sensory loss (usually with a detectable sensory level) with loss of sphincter control (rectal & urinary bladder).	3	9
Cranial nerve palsy	Facial nerve palsy, recurrent nerve palsy, oculomotor nerve palsy etc. excluding sensorineural hearing loss and ophthalmic symptoms due to inflammation	3	6
Sensory peripheral neuropathy	Sensory neuropathy resulting in glove &/or stocking distribution of sensory loss. Other causes should be excluded (e.g. idiopathic, metabolic, vitamin deficiencies, infectious, toxic, hereditary).	3	6
Motor mononeuritis multiplex	Simultaneous neuritis of single or many peripheral nerves, only scored if motor involvement. Other causes should be excluded (diabetes, sarcoidosis, carcinoma, amyloidosis).	3	9
**10. OTHER**	Other feature of active vasculitis (e.g. malaise, pulmonary hypertension, auricular chondritis etc.) - please describe		
