# Supplementary material for: Takayasu arteritis in childhood: retrospective experience from a tertiary referral centre in the United Kingdom
Source: Arthritis Res Ther. 2015 Feb 25;17(1):36. doi: 10.1186/s13075-015-0545-1 (PMC4392477; doi:10.1186/s13075-015-0545-1)
Supplement: Additional file 6: Table S1. — Treatment for 11 children with Takayasu arteritis in a UK-based centre. †In case of failed induction. Pred = oral prednisolone; mepred = methylprednisolone (30 mg/kg/day intravenously three times per day, unless stated otherwise); MTX = methotrexate (15 mg/m2wk, unless otherwise stated); CYC = cyclophosphamide; iv = intravenous; sc = subcutaneous; IVIG = intravenous immunoglobulin; PVAS = Paediatric Vasculitis Activity Score; DEI.TAK = Disease Extent Index-Takayasu; ITAS2010 = Indian Takayasu Arteritis Activity Score 2010; asp = aspirin (2 to 5 mg/kg/day); inflix = infliximab (6 mg/kg intravenously monthly unless stated otherwise); adalim = adalimumab (24 mg/m2; maximum dose 40 mg, sc every 2 weeks); NA = not applicable. For other treatments where dose is not stated, standard paediatric doses were used. [file 13075_2015_545_MOESM6_ESM.doc]

**Additional file 6: Table S1:** Treatment for 11 children with Takayasu arteritis in a UK based centre. **^┼^** In case of failed induction; Pred=oral prednisolone; mepred= methylprednisolone (iv 30mg/kg/day x3 unless stated otherwise); MTX=methotrexate (15 mg/m^2^ per week, unless otherwise stated); CYC=cyclophosphamide; iv=intravenous; sc=subcutaneous; IVIG=intravenous immunoglobulin; PVAS=Paediatric Vasculitis Activity Score; DEI.TAK=Disease Extent Index-Takayasu; ITAS-2010=Indian Takayasu Arteritis Activity Score 2010; asp= aspirin (2-5 mg/kg/day); inflix= infliximab (6 mg/kg intravenously monthly unless stated otherwise); adalim= adalimumab (24 mg/m^2^ ; max dose 40 mg, sc every 2 weeks); NA= not applicable. For other treatments where dose not stated, standard paediatric doses were used.

| **Patient**  **n°** | **Induction therapy** | **Maintenance therapy or second-line treatment^┼^** | | | | | **Biologic therapy (and concomitant immunosuppression)** | | | | | **Other treatment** | **Disease activity measures at latest follow up** | | |
| --- | --- | --- | --- | --- | --- | --- | --- | --- | --- | --- | --- | --- | --- | --- | --- |
|  |  | **Therapy** | **Months from diagnosis** | **PVAS** | **ITAS-2010** | **DEI.TAK** | **Therapy** | **Months from diagnosis** | **PVAS** | **ITAS-2010** | **DEI.TAK** |  | **PVAS** | **ITAS.2010** | **DEI.TAK** |
| 1 | Pred (1.3mg/kg/day); IVIG 2g/kg;  CYC (7 iv pulses every 4 weeks: total of 6 g/m^2^) | Pred 0.3 mg/kg; MTX  first orally then sc | 8 | 8/63 | 5/57 | 5/82 | Adalim | 9 | 4/63 | 6/57 | 1/82 | Asp  Warfarin | 0 | 0 | 0 |
| 2 | Mepred (iv 30mg/kg/day x 3 then  Pred 2mg/kg/day); MTX sc | None | NA | - | - | - | Pred 0.9 mg/kg;  MTX  Inflix  monthly (2 doses);  switched to  Adalim | 4 | 9/63 | 9/57 | 10/82 | Asp  Amlodipine  Furosemide  Captopril  Propranolol  Warfarin | 0 | 0 | 0 |
| 3 | Mepred then Pred  (0.6 mg/kg/day); CYC (7 iv pulses every 4 weeks; total of 5 g/m^2^) | MTX  sc;  pred 0.3 mg/kg/day | 8 | 2/63 | 6/57 | 4/82 | Adalim;  Pred 0.2 mg/kg/day  then | 9 | 0/63 | 4/57 | 2/82 | Asp  Clexane Furosemide Nifedipine |  |  |  |
|  |  |  |  |  |  |  | Tocilizumab  8 mg/kg 4 weekly; Pred 0.2 mg/kg/day | 14 | 2/63 | 6/57 | 4/82 |  | 4 | 10 | 5 |
| 4 | Mepred;  then pred  1mg/kg/day; IVIG 2g/kg; then  CYC (daily oral,  4 months;  5 g/m2 total) | MTX sc;  Pred 0.2 mg/kg/day | 5 | 4/63 | 8/57 | 8/82 | None | - | - | - | - | Asp  Nifedipine Propranolol | 0 | 0 | 0 |
| 5 | None | None | NA | - | - | - | None | - | - | - | - | Captopril Propranolol | 0 | 0 | 0 |
| 6 | Pred 2 mg/kg/day; AZA  (initially 2 mg/kg/day; increased to 3.7 mg/kg/day) | MTX sc;  Pred 0.75 mg/kg/day | 132 | 10/63 | 17/57 | 7/82 | Adalim  Pred 0.1 mg/kg/day | 142 | 2/63 | 4/57 | 3/82 | Amlodipine (later changed to Nifedipine)  Clonidine  Doxazosin, | 0 | 0 | 0 |
| 7 | None | None | NA | - | - | - | None | - | - | - | - | None | 7 | 9 | 7 |
| 8 | Pred  0.7 mg/kg/day; MTX sc | None | NA | - | - | - | None | - | - | - | - | Asp | 0 | 0 | 0 |
| 9 | Pred 1 mg/kg/day | None | NA | - | - | - | None | - | - | - | - | Verapamil (later changed to Nifedipine)  Atenolol | 0 | 0 | 0 |
| 10 | Pred  1 mg/kg/day; MTX sc | Pred 0.3 mg/kg/day MTX sc | NA | - | - | - | Inflix;  Pred 0.2 mg/kg/day | 24 | 4/63 | 6/57 | 4/82 | Asp  Amlodipine  Ramipril | 0 | 0 | 0 |
| 11 | Mepred;  then  pred  1mg/kg/day; MTX sc | Pred 2 mg/kg/day  CYC (2 doses iv at a total of 0.9 g/m^2^ | 7 | 11/63 | 14/57 | 15/82 | Inflix;  Pred 2mg/kg/day | 11 | 16/63 | 18/57 | 20/82 | Clonidine  Ramipril  Nifedipine Furosemide  Spironolactone  Sildenafil Epoprostenol | 21 | 18 | 20 |
