# Supplementary material for: Takayasu arteritis in childhood: retrospective experience from a tertiary referral centre in the United Kingdom
Source: Arthritis Res Ther. 2015 Feb 25;17(1):36. doi: 10.1186/s13075-015-0545-1 (PMC4392477; doi:10.1186/s13075-015-0545-1)
Supplement: Additional file 7: Table S2. — Factors associated with mortality in a cohort of 11 children with Takayasu arteritis seen in a UK tertiary referral centre. Fisher’s exact test was used for comparison between groups. PVDI = Paediatric Vasculitis Damage Index [19]. P-values <0.05 (two-sided) were considered significant. [file 13075_2015_545_MOESM7_ESM.doc]

**Additional file 7: Table S5:** Factors associated with mortality in a cohort of 11 children with Takayasu arteritis seen in a UK tertiary referral centre. Fishers exact test was used for comparison between groups. PVDI=paediatric vasculitis damage index [19]. P values< 0.05 (two sided) were considered significant.

|  | **Dead (n)** | **Alive (n)** | **P value** |
| --- | --- | --- | --- |
| Age at disease onset  < 5 years  > 5 years | 3  0 | 1  7 | **0.0242** |
| Time to diagnosis  >12 months  <12 months | 2  1 | 3  5 | 0.545 |
| Male  Female | 1  2 | 3  5 | 1 |
| Cardiac involvement  Yes  No | 3  0 | 3  5 | 0.18 |
| Endovascular surgery within 12 months of diagnosis  Yes  No | 2  1 | 3  5 | 0.545 |
| Biologic therapy  Yes  No | 2  1 | 4  4 | 1 |
| PVDI (excluding items no longer present) >3  Yes  No | 3  0 | 1  7 | **0.0242** |
